# Identification and validation of radiomic features from computed tomography for preoperative classification of neuroblastic tumors in children

**DOI:** 10.1186/s12887-023-04057-3

**Published:** 2023-05-24

**Authors:** Lian Zhao, Liting Shi, Shun-gen Huang, Tian-na Cai, Wan-liang Guo, Xin Gao, Jian Wang

**Affiliations:** 1 Radiology Department, Children’s Hospital of Soochow University, Suzhou, Jiangsu 215025 China; 2 Division of Life Sciences and Medicine, School of Biomedical Engineering (Suzhou), University of Science and Technology of China, Hefei, Anhui 230026 China; 3 Suzhou Institute of Biomedical Engineering and Technology, Chinese Academy of Sciences, Suzhou, Jiangsu 215163 China; 4 Pediatric Surgery Department, Children’s Hospital of Soochow University, Suzhou, Jiangsu 215025 China; 5 Jinan Guoke Medical Engineering and Technology Development Co., Ltd, Jinan, Shandong 250101 China

**Keywords:** Neuroblastic tumors, Radiomics, Computed tomography, Ganglioneuroma, Ganglioneuroblastoma, Neuroblastoma

## Abstract

**Background:**

To identify radiomic features that can predict the pathological type of neuroblastic tumor in children.

**Methods:**

Data on neuroblastic tumors in 104 children were retrospectively analyzed. There were 14 cases of ganglioneuroma, 24 cases of ganglioneuroblastoma, and 65 cases of neuroblastoma. Stratified sampling was used to randomly allocate the cases into the training and validation sets in a ratio of 3:1. The maximum relevance–minimum redundancy algorithm was used to identify the top 10 of two clinical features and 851 radiomic features in portal venous–phase contrast-enhanced computed tomography images. Least absolute shrinkage and selection operator regression was used to classify tumors in two binary steps: first as ganglioneuroma compared to the other two types, then as ganglioneuroblastoma compared to neuroblastoma.

**Results:**

Based on 10 clinical-radiomic features, the classifier identified ganglioneuroma compared to the other two tumor types in the validation dataset with sensitivity of 100.0%, specificity of 81.8%, and an area under the receiver operating characteristic curve (AUC) of 0.875. The classifier identified ganglioneuroblastoma versus neuroblastoma with a sensitivity of 83.3%, a specificity of 87.5%, and an AUC of 0.854. The overall accuracy of the classifier across all three types of tumors was 80.8%.

**Conclusion:**

Radiomic features can help predict the pathological type of neuroblastic tumors in children.

## Introduction

Neuroblastic tumors, which originate from sympathetic natural crest cells in the early stage of neural development [1], are the most frequent type of extracranial solid tumors in children [2]. Although such tumors can occur in any part of the sympathetic nervous system, most often they include the abdominal spinal sympathetic ganglia (in 60% of cases) and adrenal gland (in 30% of cases) [3].

Three types of neuroblastic tumors have been described, which differ significantly in the course of their disease and in optimal treatment [4]: ganglioneuroma, ganglioneuroblastoma, and neuroblastoma. Neuroblastoma, which is highly malignant, contains undifferentiated neuroblasts [5]. This tumor type accounts for 10% of malignant tumors and 15% of tumor-related mortality in children. Ganglioneuroblastoma, which is considerably less malignant than neuroblastoma, contains neuroblasts, glial fibers, proliferative nerve sheath cells, and ganglion cells in different degrees of differentiation. Ganglioneuroma is benign.

To optimize treatment, it is necessary to accurately determine the type of neuroblastic tumors in children [6]. At present, tumors are assigned to one of the three types based on pathological examination of the biopsies, which is invasive and carries a risk of complications [7]. Moreover, such typing depends on the experience of the clinician and the exact location of the biopsy in the heterogeneous tumor tissue. A more objective and noninvasive approach could avoid these shortcomings.

With the constant development and cross-integration of medical imaging, computer science, informatics, and other disciplines, traditional image diagnostics is undergoing a new round of change. Radiomics realizes high-throughput feature extraction, analysis, and quantification of image data using automated algorithms, thus providing new interpretations of the potential features of the images. Radiomics can extract microscopic details of a large number of tumor lesions that are difficult to identify with human eyes and may quantify their internal subtle structures to obtain a set of image markers related to the disease. It has been proven that many radiomic features are useful for predicting the stage of an abdominal tumor, evaluation of effectiveness, and prognosis [8]. Computed tomography (CT) is widely used to detect and diagnose neuroblastic tumors in the clinical environment, and certain radiomic features from images obtained using CT or other methods are useful in the diagnosis and typing of various diseases [9–12]. CT is noninvasive and effectively records the heterogeneity of the tumor [13, 14], so it is important to determine whether we could identify CT-based radiomic features for typing neuroblastic tumors in children. Based on this idea, such features have proven useful in predicting the proliferation of MYCN in neuroblastoma and ganglioneuroblastoma [15].

Therefore, here we have defined and validated a radiomic classifier based on contrast-enhanced CT to distinguish the three types of neuroblastic tumors.

## Methods and materials

### Patients

Data were analyzed retrospectively for all patients who had been treated for pathology-confirmed neuroblastic tumors of any type at the Children’s Hospital of Soochow University (Suzhou, China) between January 2015 and December 2021. This study was approved by the institutional review board of the Children’s Hospital of Soochow University. Written informed consent was provided by the parents or legal guardians of the children.

### CT and image segmentation

Contrast-enhanced CT was conducted using a 64-slice system (GE Optima CT660, GE Healthcare, Optima 660; GE Medical System, Milwaukee, WI, USA). The scanning parameters were as follows: tube voltage, 70–120 kVp; tube current, 10–1041 mA; rotation time, 0.35–4.53 s; pixel spacing, 0.32–0.73 mm; slice thickness, 5 mm; and slice interval, 5 mm.

Patients who could not cooperate were sedated with 10% chloral hydrate (0.5 ml/kg) for a total of no more than 10ml. Subjects whose CT image motion artifacts were too large and could influence clinical diagnosis were again given an enhanced CT scan at the scheduled time.

**Fig. 1:**
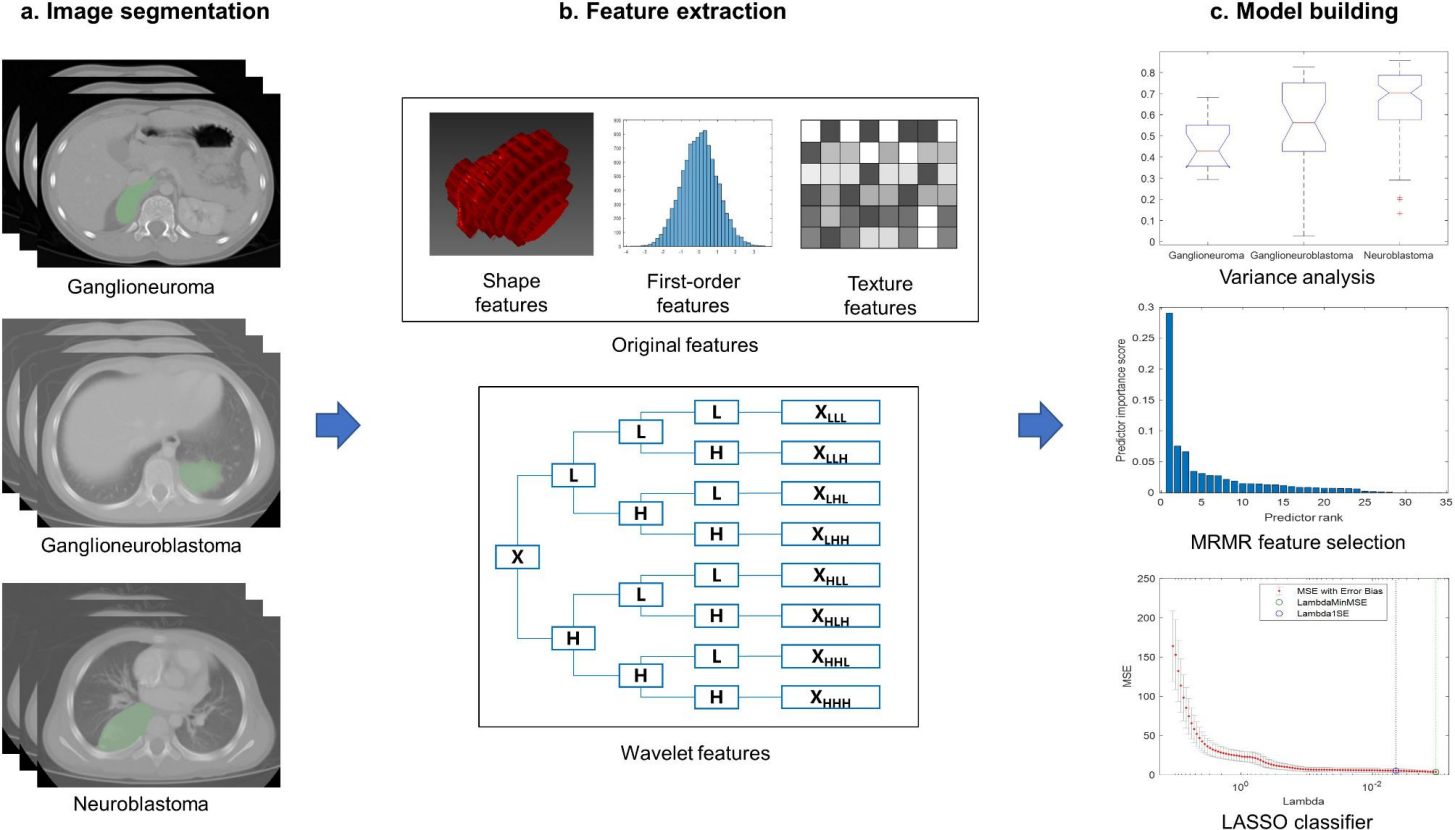
Workflow in this study. Tumors in portal venous–phase contrast-enhanced computed tomography images were manually contoured by an experienced radiologist, and radiomic features were analyzed within the tumor region. Variance analysis and the maximum relevance–minimum redundancy (MRMR) algorithm were used to selected the top 10 diagnostic features. These features were then used to develop a classifier based on least absolute shrinkage and selection operator (LASSO) regression

Using the 3D Slicer software (https://www.slicer.org/), a radiologist with 5 years of experience manually described all the tumors in the CT images of the portal vein phase (Fig. 1a). Segmentation was supervised and confirmed by a radiologist with 10 years of experience.

### Image preprocessing and feature extraction

Portal venous–phase contrast-enhanced CT images, which were resampled to a voxel size of 0.5 mm ⋅ 0.5 mm ⋅ 5 mm to reduce inter-subject variation in the reconstruction parameters, were re-binned using a bin width of 20 to minimize bias due to sparsely populated matrices [16–18].

The following four types of radiomic features were extracted from the images using the Pyradiomics package (https://pyradiomics.readthedocs.io/en/latest/index.html) in Python 3.6: shape features, first-order features, texture features, and wavelet features (Fig. 1b) [9, 19]. Shape features, such as sphericity and compactness, describe the three-dimensional shape and size of the tumor. First-order features, such as kurtosis and median, are first-order statistics that quantify the distribution of voxel intensities. Texture features, which characterize the spatial distribution of voxel intensities in a neighborhood, are calculated based on the gray level co-occurrence matrix (“glcm”), gray level dependence matrix (“gldm”), gray level run length matrix (“glrlm”), gray level size zone matrix (“glszm”), and neighboring gray tone difference matrix (“ngtdm”). Wavelet features are the first-order and texture features that emerge after the wavelet filter is applied to the images. Finally, the extracted features were normalized using a robust method that scaled the measured parameters and excluded outliers based on the observed means and standard deviations [20].

### Feature selection

Stratified sampling was used to randomly distribute the data into training and validation sets in a 3:1 ratio while achieving a similar distribution of the three tumor types between the two sets.

Next, the potentially diagnostic radiomic features were selected from all of the features in the training set (Fig. 1c). In the selection process, the radiomic features were compared among the three tumor types using one-way analysis of variance (ANOVA) for normally distributed variables, or the Kruskal–Wallis test for variables with a skewed distribution. The radiomics features associated with P < 0.05 and two clinical features (age and gender) were ranked using the maximum relevance–minimum redundancy algorithm [13, 14, 21], and the top 10 features were selected to create a classifier.

### Derivation of the classifier

The final classifier was defined using least absolute shrinkage and selection operator (LASSO) regression (Fig. 1c) [14, 21, 22]. LASSO regularization of linear models of the input variables (radiomic features) and the response variable (tumor type) generated least-squares regression coefficients, which were then used to build the LASSO classifier. The model was optimized based on the minimum five-fold cross-validated mean squared error (MSE).

The final model for classifying the tumors into one of the three types proceeded through two binary classifications: first, ganglioneuroma compared to the other two types of tumors, followed by ganglioneuroblastoma compared to neuroblastoma. To compare the classification performance of conventional clinical features and radiomics features, two models were developed, one based solely on clinical data (clinical model) and the other on the same clinical data together with the top radiomic features mentioned in Sect. 2.4 (clinical-radiomics model).

### Assessment of the classifier’s performance

The performance of the model in classifying the tumors into one type compared to the other two types was evaluated in terms of sensitivity, specificity, and the area under the receiver operating characteristic curve (AUC). To assess the overall accuracy, the number of correctly classified subjects with any tumor type was divided by the total number of subjects. To evaluate the accuracy for each type of tumor, the number of correctly classified subjects with that tumor type was divided by the total number of subjects with that type of tumor.

## Results

### Patient characteristics

The analysis included 103 subjects, of whom 65 had neuroblastoma, 24 had ganglioneuroblastoma, and 14 had ganglioneuroma based on pathological data (Table 1).

**Table 1:** Clinicodemographic and prognostic characteristics of the 103 pediatric patients with neuroblastic tumors in this study

Characteristic	Type of neuroblastic tumor*		
	Ganglioneuroma	Ganglioneuroblastoma	Neuroblastoma
Sex			
Male	8	13	38
Female	6	11	27
Age, months	7.36 (± 3.77)	4.38 (± 2.61)	2.38 (± 2.49)
International neuroblastoma stage**			
I	14	12	6
II	0	3	3
III	0	3	13
IV	0	6	36
IVs	0	0	6
Event-free survival			
Yes	14	21	43
No	0	3	22
Overall survival			
Yes	14	23	52
No	0	1	13

Values are n or mean (SD).

* Based on the International Neuroblastoma Pathology Classification (INPC system)

** Based on the International Neuroblastoma Staging System (INSS)

### Feature selection

**Fig. 2:**
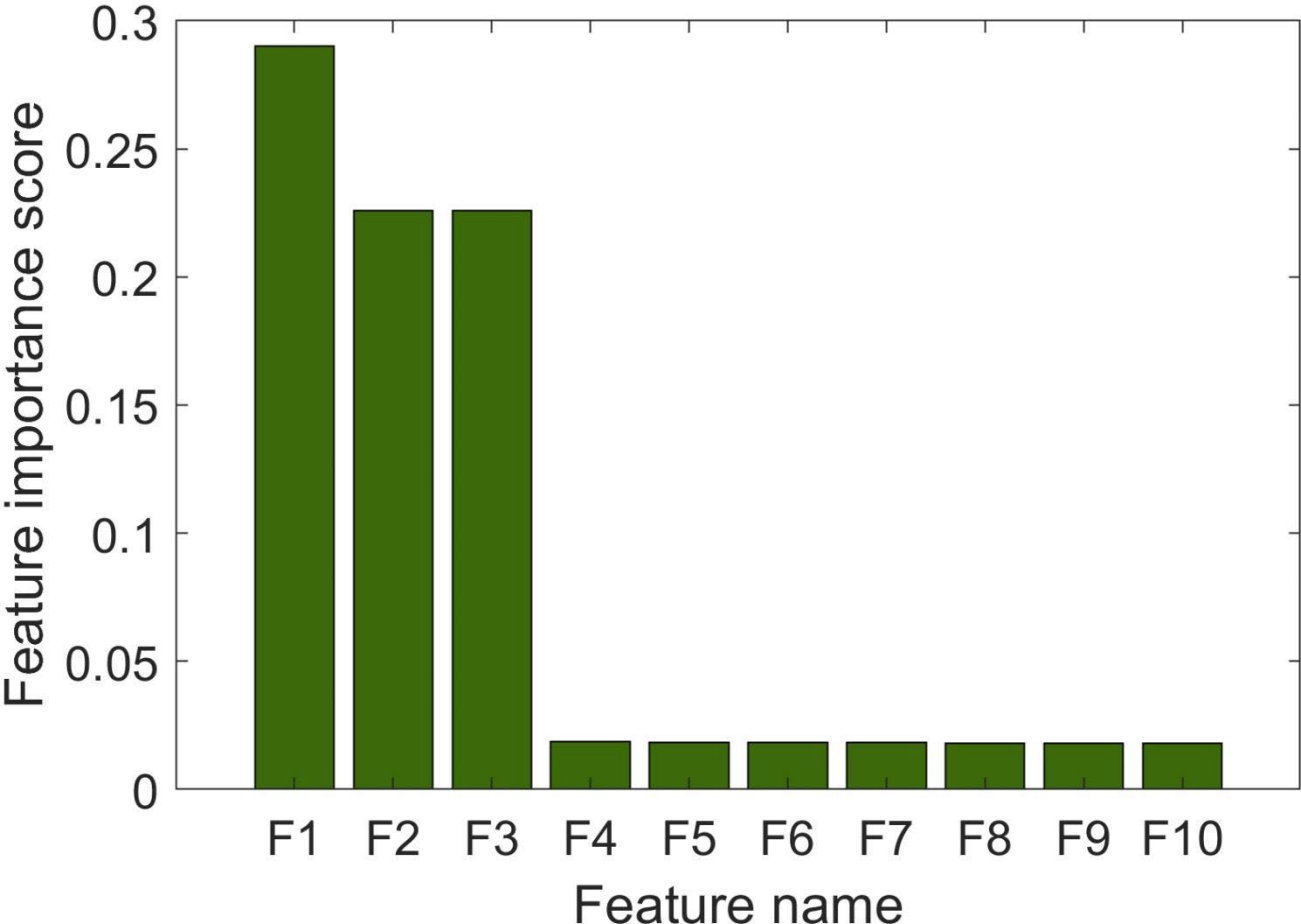
The importance scores of the top 10 features chosen by the maximum relevance-minimum redundancy algorithm. F1, original_glcm_Correlation; F2, wavelet-HLH_glszm_SmallAreaEmphasis; F3, wavelet-LHL_glcm_Imc1; F4, wavelet-LLH_glszm_LowGrayLevelZoneEmphasis; F5, wavelet-LLL_glcm_Imc2; F6, age; F7: original_glcm_Imc1; F8, original_glcm_Imc2; F9, wavelet-LLL_glcm_Correlation; F10, wavelet-HLH_glszm_SizeZoneNonUniformityNormalized.

For each subject, data were extracted for 851 shape, first-order, texture, and wavelet features and two clinical features (age and gender), and the following top 10 features emerged from the maximum relevance–minimum redundancy algorithm: “age,” “original_glcm_Correlation,” “wavelet-HLH_glszm_SmallAreaEmphasis,” “wavelet-LHL_glcm_Imc1,” “wavelet-LLH_glszm_LowGrayLevelZoneEmphasis,” “wavelet-LLL_glcm_Imc2,” “original_glcm_Imc1,” “original_glcm_Imc2,” “wavelet-LLL_glcm_Correlation,” and “wavelet-HLH_glszm_SizeZoneNonUniformityNormalized” (Fig. 2). In this way, three of the top 10 features were texture features, while six were wavelets.

### Model performance

**Fig. 3:**
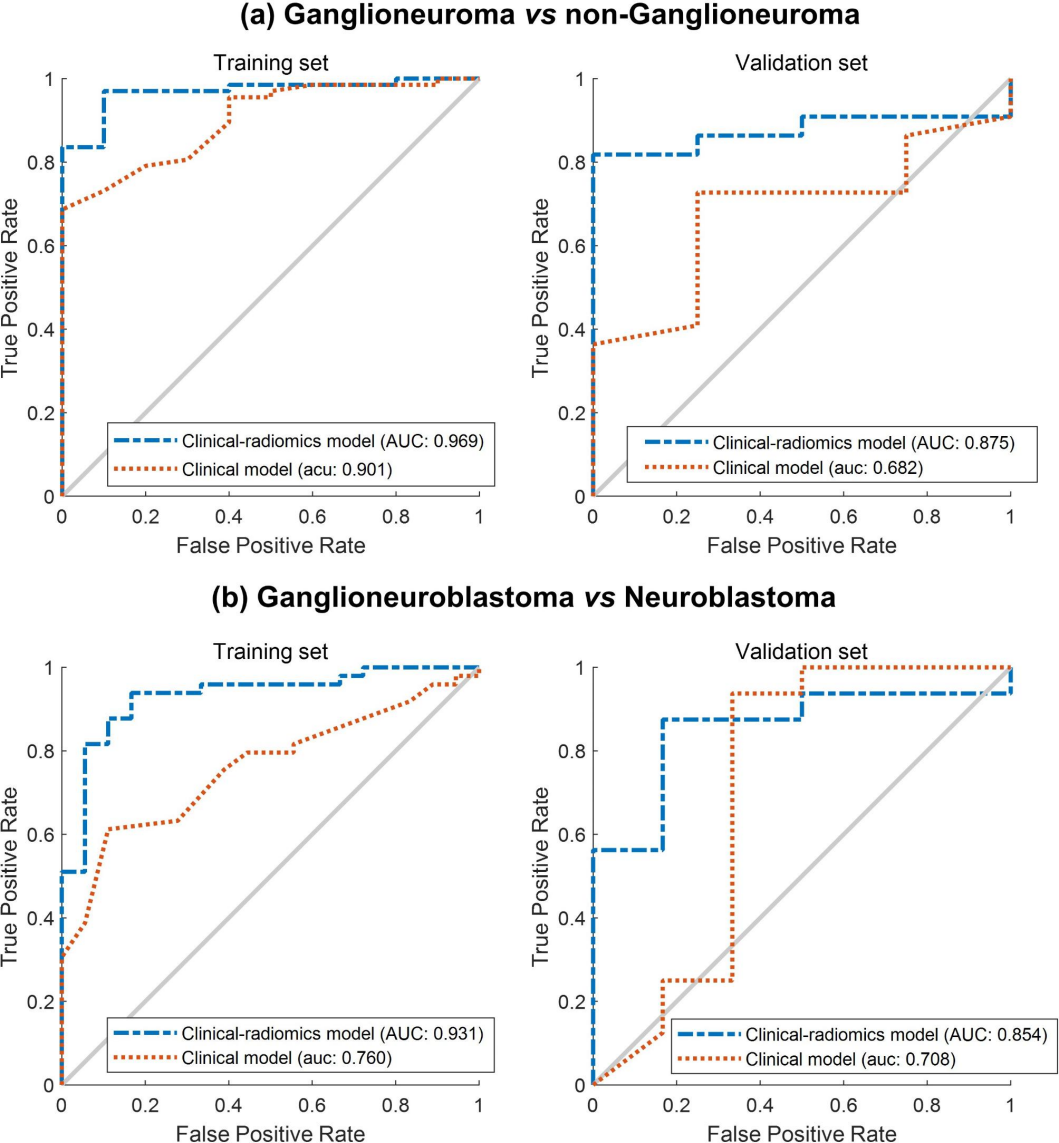
Receiver operating characteristic curves showing the performance of the classifiers based only on clinical data and on the combination of clinical data and radiomic features at each binary classification step: (a) ganglioneuroma compared to non-ganglioneuroma, (b) ganglioneuroblastoma compared to neuroblastoma. Results are shown separately for the training set (left) and the validation set (right). AUC, area under the curve

By combining radiomic features and clinical data, the classifier showed a balanced accuracy of 80.8% in the validation set (Table 2), compared to only 46.2% for the classifier that included only clinical data (Table 3). In the training set, the radiomic-clinical classifier distinguished ganglioneuroma from the other two tumor types with a sensitivity of 90.0%, a specificity of 94.0%, and an AUC of 0.969 (Table 2; Fig. 3). The corresponding values in the validation set were 100.0%, 81.8%, and 0.875. In the training set, the radiomic-clinical classifier distinguished ganglioneuroblastoma from neuroblastoma with a sensitivity of 83.3%, a specificity of 91.8%, and an AUC of 0.931. The corresponding values in the validation set were 83.3%, 87.5%, and 0.854.

**Table 2:** Performance of the radiomic-clinical classifier for distinguishing the three types of neuroblastic tumor

Classification step	Tumor Type	Training set					Validation set				
		Accuracy	Sensitivity	Specificity	AUC	Accuracy		Sensitivity	Specificity		AUC
Individual binary classifications	GN vs. non-GN	93.5%	90.0%	94.0%	0.969	84.6%		100%	81.8%	0.875	
	GNB vs. NB	89.6%	83.3%	91.8%	0.931	86.4%		83.3%	87.5%	0.854	
		**GN**	**GNB**	**NB**	**Overall accuracy**	**GN**		**GNB**	**NB**	**Overall accuracy**	
Three-way classification	GN vs. GNB vs. NB	90.0%	66.7%	91.8%	85.7%	100%		50%	87.5%	80.8%	

AUC, area under the receiver operating characteristic curve; GN, ganglioneuroma; GNB, ganglioneuroblastoma; NB, neuroblastoma

**Table 3:** The performance of the clinical model for classifying three tumors

Classification models	Tumor Type	Training set					Validation set				
		Accuracy	Sensitivity	Specificity	AUC	Accuracy		Sensitivity	Specificity		AUC
Binary classification model	Ganglioneuroma vs. non-Ganglioneuroma	75.3%	90.0%	73.1%	0.901	73.1%		75%	72.7%	0.682	
	Ganglioneuroblastoma vs. Neuroblastoma	73.1%	55.6%	79.6%	0.760	59.1%		66.7%	56.3%	0.708	
		**Ganglioneuroma**	**Ganglioneuroblastoma**	**Neuroblastoma**	**Overall accuracy**	**Ganglioneuroma**		**Ganglioneuroblastoma**	**Neuroblastoma**	**Overall accuracy**	
*Three classification model*	*Ganglioneuroma vs. Ganglioneuroblastoma vs. Neuroblastoma*	*90.0%*	*11.1%*	*79.6%*	*64.9%*	*75.0%*		*0%*	*56.3%*	*46.2%*	

**Fig. 4:**
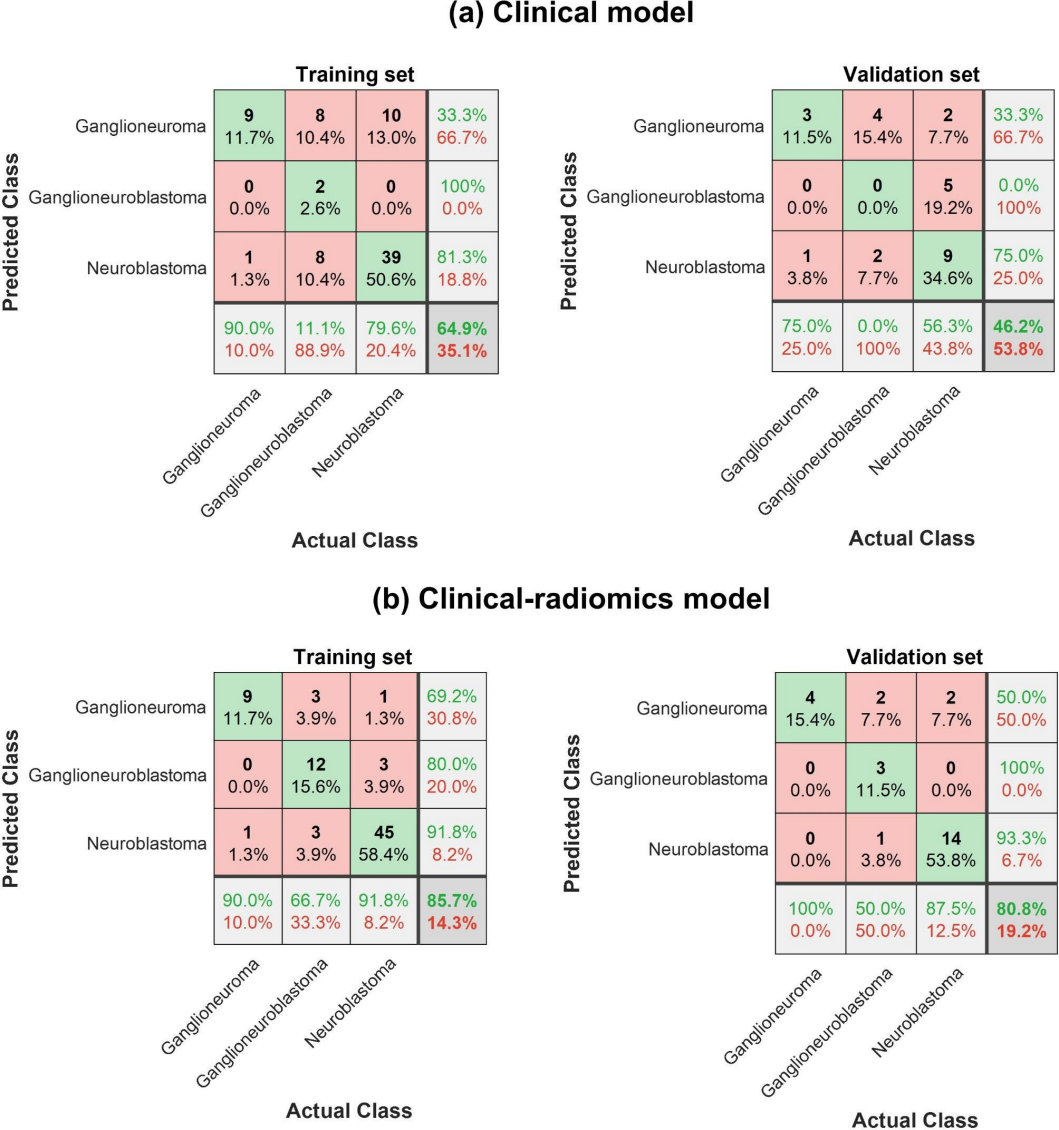
Confusion matrices showing the performance of the classifiers based on (a) only clinical data and (b) the combination of clinical data with radiomic features. Columns indicate the number of subjects who actually belong to the class, while rows indicate the number of subjects predicted to belong to the class. The percentages around the edge of the matrices refer to the accuracy (green text) and error rates (red text) of each column, each row, and the whole matrices (lower right corner)

The overall accuracy of the radiomic-clinical classifier was 85.7% in the training set and 80.8% in the validation set (Fig. 4).

## Discussion

Although the three types of neuroblastic tumors occur in similar locations and cause similar symptoms in children, they differ substantially in prognosis and optimal treatment [6]. Currently, the tumors are typed on the basis of pathological examination of biopsies, but this is invasive and carries the risk of complications [7]. Here, we provide evidence that noninvasive tumor assessment based on clinical data and preoperative CT can predict the type of tumor and therefore help guide treatment.

The overall accuracy of our radiomic-clinical classifier was 80.8% in the validation set, suggesting the need for further optimization. Nevertheless, the model was reasonably accurate at identifying ganglioneuroma, showing sensitivity of 100.0% and specificity of 81.8%. Model performance can be improved by using large samples from multiple centers and by exploring machine learning algorithms other than LASSO. Taking into account our small, single-center sample, the radiomic-clinical model should be tested for generalizability and further optimized on the basis of larger samples from multiple centers.

We selected the radiomic features for our classifier using the maximum relevance–minimum redundancy algorithm, which has increasingly been used in radiomic studies since its first description in 2005 [23]. Unlike earlier methods for feature selection, this algorithm takes into account the correlation between features, thereby reducing the risk of false positives and improving the generalizability of the model [24, 25].

Our results emphasize the usefulness of texture features in differentiating the types of neuroblastic tumors, most likely reflecting that such features can capture the heterogeneity of the tumor structure [26]. Among the three tumor types, 10 features were distinguished, the overall accuracy of which was 85.7% in the training set and 80.8% in the validation set, and the performance was even better in the two binary classifications within the model. Thus, the ability to distinguish between neuroblastoma, ganglioneuroma, and ganglioneuroblastoma depends on the identification of differences in the structure of tissues.

Six of the 10 features extracted in the present study belong to spatial gray level co-occurrence matrix (GLCM) features. Previous studies have shown that GLCM features are helpful in the pretreatment prediction of pathological complete response to no special type(NST) in breast cancer [27]. GLSZM texture features have been useful in differentiating between two different tumors in studies using CT imaging omics to distinguish between pelvic rhabdomyosarcoma and yolk cystoma in children [28]. In the present study, GLCM and GLSMZ were useful in predicting the pathological type of neuroblastic tumors in children, suggesting that the texture characteristics of different tumor types differ. At the same time, most of the valuable features in the present study were based on wavelet and LoG. Both the wavelet and the LoG are high-order statistical methods for placing filter grids on the image to extract repetitive or nonrepetitive patterns, both of which help reveal more valuable information that is not visible in the lesion [8].

The present study determines the feasibility of using contrast-enhanced CT to diagnose neuroblastic tumors before surgery. Further validation and optimization studies may help develop an accurate, noninvasive tool that can guide treatment.

## Data Availability

The datasets used and/or analyzed during the current study available from the corresponding author on reasonable request.
